# Investigation of the long-term effects of high-potency antiviral agents on aspartate aminotransferase-to-platelet ratio index and fibrosis index based on four factors: five-year outcomes in hepatitis B e-antigen-negative chronic hepatitis B patients

**DOI:** 10.1590/1806-9282.20250374

**Published:** 2025-10-17

**Authors:** Mustafa Arslan, Ahmet Mert Cavnar, Şirin Çetin

**Affiliations:** 1 Amasya University, Sabuncuoğlu Şerefeddin Training and Research Hospital, Department of İnfectious Diseases and Clinical Microbiology – Amasya, Turkey.; 2 Sabuncuoğlu Şerefeddin Training and Research Hospital, Department of İnfectious Diseases and Clinical Microbiology – Amasya, Turkey.; 3 Amasya University, Department of Biostatistics – Amasya, Turkey.

**Keywords:** Fibrosis, Chronic hepatitis B, Efficacy, Entecavir, Tenofovir alafenamide, Tenofovir disoproxil fumarate

## Abstract

**OBJECTIVE:**

The aim of this study was to investigate the long-term effects of tenofovir alafenamide, tenofovir disoproxil fumarate, and entecavir on fibrotic burden and to compare the virological clearance and biochemical improvement times achieved with these drugs.

**METHODS:**

The study was designed with treatment-naive, hepatitis B e-antigen-negative chronic hepatitis B patients who started tenofovir alafenamide, tenofovir disoproxil fumarate, or entecavir at a tertiary care hospital. The aspartate aminotransferase-to-platelet ratio index and the fibrosis index based on four factors were used to determine the fibrotic burden.

**RESULTS:**

Age, gender, baseline aspartate aminotransferase-to-platelet ratio index and fibrosis index based on four factors values, and fibrosis grades obtained by the biopsy of patients treated with tenofovir alafenamide (n=45), tenofovir disoproxil fumarate (n=65), and entecavir (n=56) were similar (p>0.05 for all). Significant fibrosis regression was observed during the 5-year antiviral therapy period in the tenofovir alafenamide, tenofovir disoproxil fumarate, and entecavir groups.

**CONCLUSION:**

Significant regression in aspartate aminotransferase-to-platelet ratio index and fibrosis index based on four factors was observed during tenofovir alafenamide, tenofovir disoproxil fumarate, and entecavir therapy within the first 2 years, and this regression remained stable up to the 5th year. None of the three drugs showed superiority over each other in terms of the pattern of fibrosis regression reflected by aspartate aminotransferase-to-platelet ratio index and fibrosis index based on four factors, average virological response time, and biochemical improvement time.

## INTRODUCTION

Hepatitis B virus (HBV) is an infectious agent that infects human liver cells and causes inflammation in the liver, leading to serious issues such as chronic infection, liver cirrhosis, or hepatocellular carcinoma (HCC). For many years, liver biopsy has been considered the gold standard in staging fibrosis. However, liver biopsy has several limitations^1^. The procedure is invasive and painful^2^, carries rare but potentially life-threatening complications^3^, and is prone to sampling errors^4,5^. Consequently, these limitations associated with biopsy have prompted research into non-invasive methods. For the non-invasive assessment of fibrosis in chronic hepatitis B (CHB) patients, various methods have been proposed, including serum markers such as the aspartate aminotransferase-to-platelet ratio index (APRI) and the fibrosis index based on four factors (FIB-4). The APRI and FIB-4 scores are non-invasive methods that can diagnose advanced fibrosis and cirrhosis in CHB patients with high accuracy^6^. According to the World Health Organization's guidelines on the management of CHB, for the diagnosis of significant fibrosis (fibrosis stages ≥F2), the sensitivity of the APRI is 78%, while its specificity is 92%^7^. The FIB-4 cutoff value of ≥1.45 distinguishes moderate fibrosis from severe fibrosis with a sensitivity of 71% and a specificity of 73%^8^. Non-invasive fibrosis tests can predict significant fibrosis and cirrhosis and help in selecting the best patients for HBV infection treatment. The regression of fibrosis with long-term tenofovir disoproxil fumarate (TDF) and entecavir (ETV) therapy has been demonstrated biochemically, virologically, and histologically^9,10^. There is no study in the literature that simultaneously compares the changes in APRI and FIB-4 values following long-term treatment with tenofovir alafenamide (TAF), TDF, and ETV. We aimed to investigate the effects of long-term (for 5 years) antiviral therapy on non-invasive fibrosis tests and to evaluate the roles of APRI and FIB-4 values in monitoring long-term prognosis in hepatitis B e-antigen (HBeAg)-negative CHB patients.

## METHODS

### Patients

Our study was conducted retrospectively with CHB patients followed up at Amasya University Faculty of Medicine Sabuncuoğlu Serefeddin Education and Research Hospital between January 2014 and October 2024. The 5-year data of the selected patients from the start of treatment were evaluated. APRI and FIB-4 were calculated annually for 5 years, starting from the beginning of treatment.

The cases in each of the three treatment groups were included in the study based on the following criteria:

- Patients who had a liver biopsy before treatment with a diagnosis of CHB
- Patients who were HBeAg-negative and anti-HBe antibody-positive
- Treatment-naive patients
- Over 18 years of age
- Regular follow-up for 5 years
- Treated with either TAF, TDF, or ETV for at least 5 years

### Calculation of non-invasive fibrosis scores

FIB-4 index: age (year)×aspartate aminotransferase (AST) (U/L)/(platelet count [10^9^/L]×[alanine aminotransferase (ALT)] 1/2 [U/L])^11^.

APRI score: AST/upper reference limit value of AST/platelet count (10^9^/L)×100^12^.

### Virological and biochemical response measurements

In the evaluation of virological and biochemical responses, the median times to HBV DNA levels becoming undetectable and ALT normalization were used.

### Statistical analysis

Statistical analyses were performed using the IBM SPSS version 23.0 (SPSS Inc., Chicago, IL, USA) software package and the SAS software package (v9.4; SAS Institute, Cary, NC, USA). Categorical variables were expressed as numbers and percentages. Differences between groups were analyzed using the Student's t-test for normally distributed data and the Mann-Whitney U test for non-normally distributed data. For comparisons of more than two groups, the one-way analysis of variance (ANOVA) was used for normally distributed data and the Kruskal-Wallis test was used for non-normally distributed data. The chi-square test was used for percentage comparisons of categorical variables. ALT, AST, platelet count, and APRI and FIB-4 scores at six different time points (baseline, and at the end of the 1st, 2nd, 3rd, 4th, and 5th years of treatment) were compared using Friedman and one-way ANOVA tests. Changes in the APRI and FIB-4 scores were analyzed using the MIXED model in the SAS software package. The fixed effects in APRI and FIB-4 scores and the changes shown by the treatment over time were also examined using the MIXED model, followed by post-hoc analyses. A p<0.05 was considered statistically significant.

## RESULTS

### Demographic characteristics

Of the 166 patients included in the study, 105 (63.3%) were male and 61 (36.7%) were female. The average age of all patients was 51.82±11.07 years. Among the patients, 45 were treated with TAF, 65 with TDF, and 56 with ETV. The age and gender characteristics were similar across the three groups (p>0.05).

### Baseline biochemical, virological, and histopathological characteristics

The mean HBV DNA levels for the treatment groups were 5.4±3.1 log_10_IU/mL, 5.2±2.8 log_10_IU/mL, and 5.5±2.0 log_10_IU/mL for TDF, TAF, and ETV, respectively. The three groups were statistically similar in terms of baseline mean ALT values and viral load (p=339). The virological, biochemical, and histopathological data of the patients are shown in Table 1.

**Table 1:** Baseline virological, biochemical, and histopathological characteristics.

	All patients (n=166)	TAF group (n=45; 27.1%), mean±SD (min–max)	TDF group (n=65; 39.2%), mean±SD (min–max)	ETV group (n=56; 33.7%), mean±SD (min–max)	p-value
Baseline HBV DNA (log_10_IU/mL) (min–max)	5.3±2.4 (2.2–26.2)	5.4±3.1 (2.2–13.8)	5.2±2.8 (3.1–10.9)	5.3±1.7 (3.3–26.2)	0.348*
Baseline AST (U/L) (min–max)	95.55±39.8 (32–231)	96.04±43.5 (32–221)	97.4±37.2 (38–231)	87.6±41.9 (41–220)	0.382*
Baseline ALT (U/L) (min–max)	106.74±48.2 (29–288)	105.33±45.9 (29–225)	109.9±49.4 (38–288)	96.6±53.0 (35–238)	0.339*
Baseline platelet count (K/μL) (min–max)	176±38 (76–271)	170±30 (87–266)	177±40 (76–271)	180±36 (112–236)	0.413**
HAI average (min–max)	8.51±1.5 (3–14)	8.64±1.6 (3–14)	8.58±1.8 (5–11)	8.45±1.8 (4–12)	0.84**
Fibrosis average (min–max)	2.62±0.9 (0–5)	2.67±1.0 (0–5)	2.57±1.1 (0–5)	2.55±1.0 (0–5)	0.853**
Cirrhosis n (%)	34 (20.5)	10 (22.2)	13 (20)	11 (19.6)	0.122
Baseline APRI score, average (min–max)	1.38±0.74 (0.58–5.1)	1.42±0.75 (0.58–3.68)	1.39±0.84 (0.6–5.1)	1.34±0.71 (0.58–5.1)	>0.05
Baseline FIB-4 index average (min–max)	3.20±1.71 (1.27–8.72)	3.17±1.77 (0.93–8.71)	3.24±1.72 (1.27–8.72)	3.21±1.48 (1.27–7.62)	>0.05

HBV: hepatitis B virus; AST: aspartate aminotransferase; ALT: alanine aminotransferase; HAI: histological activity index; TAF: tenofovir alafenamide; SD: standard deviation; TDF: tenofovir disoproxil fumarate; ETV: entecavir; APRI: aspartate aminotransferase-to-platelet ratio index; FIB-4: fibrosis index based on four factors.

* Kruskal-Wallis test was applied.

** One-way ANOVA test was applied.

### Baseline non-invasive indicators

There was no statistically significant difference in baseline APRI and FIB-4 scores between the cumulative patient population and the treatment groups (both p>0.05) (Table 1).

### Changes in non-invasive fibrosis markers during antiviral therapy

**Figure 1:**
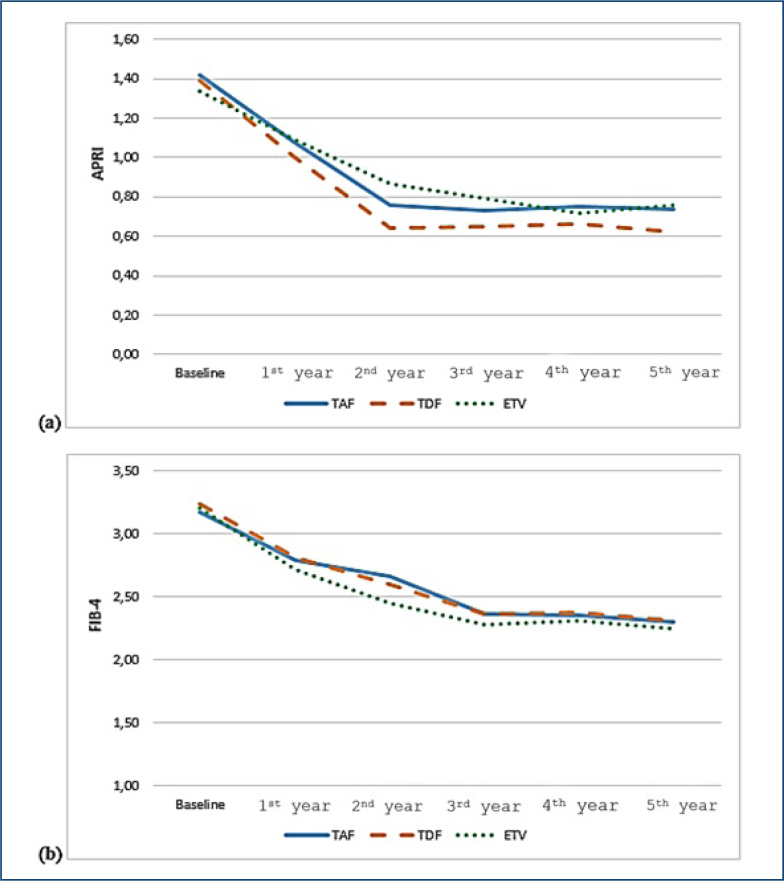
Effects of tenofovir alafenamide, entecavir, and tenofovir disoproxil fumarate on fibrosis regression. **(a)** Aspartate aminotransferase-to-platelet ratio index score and **(b)** fibrosis index based on four factors.

APRI and FİB-4 values measured at all time points were significantly lower than the baseline APRI and FİB-4 values (all p<0.0001). The APRI score and FİB-4 index showed a decrease until the 2nd year with treatment in all patients and separately in the treatment arms, and plateaued between the 2nd year and the 5th year (Table 2 and Figure 1).

**Table 2:** Yearly changes in aspartate aminotransferase-to-platelet ratio index scores and fibrosis index based on four factors.

		Baseline, mean±SD (min–max)	1st year, mean±SD (min–max)	2nd year, mean±SD (min–max)	3rd year, mean±SD (min–max)	4th year, mean±SD (min–max)	5th year, mean±SD (min–max)	p-value
APRI								
	All patients	1.38±0.74 (0.58–5.1)	1.03±0.58 (0.3–4.9)	0.77±0.58 (0.3–4.5)	0.74±0.66 (0.22–3.5)	0.72±0.40 (0.22–3.51)	0.73±0.49 (0.29–4.3)	**<0.0001**
	TAF group	1.42±0.75 (0.58–3.68)	1.08±0.68 (0.30–3.4)	0.76±054 (0.31–3.06)	0.73±0.73 (0.3–3.03)	0.75±0.23 (0.31–3.04)	0.74±0.43 (0.29–3.07)	**<0.0001**
	TDF group	1.39±0.84 (0.6–5.1)	1.00±0.71 (0.3–4.9)	0.64±0.55 (0.3–4.5)	0.65±0.54 (0.22–3.5)	0.66±0.51 (0.22–3.5)	0.62±0.49 (0.3–4.3)	**<0.0001**
	ETV group	1.34±0.71 (0.58–5.1)	1.09±0.66 (0.45–4.75)	0.87±0.55 (0.31–3.52)	0.79±0.55 (0.32–3.5)	0.72±0.67 (0.3–3.51)	0.76±0.55 (0.3–3.57)	**<0.0001**
FIB-4								
	All patients	3.20±1.71 (1.27–8.72)	2.72±1.40 (0.75–7.25)	2.57±1.38 (0.7–6.43)	2.31±1.20 (0.63–6.38)	2.36±1.23 (0.62–6.06)	2.30±1.27 (0.61–6.3)	**<0.0001**
	TAF group	3.17±1.77 (0.93–8.71)	2.79±1.58 (0.75–7.06)	2.66±1.46 (0.70–6.41)	2.37±1.26 (0.63–6.38)	2.35±1.38 (0.62–6.42)	2.30±1.31 (0.61–6.30)	**<0.0001**
	TDF Group	3.24±1.72 (1.27–8.72)	2.81±1.45 (0.94–7.25)	2.60±1.26 (0.84–6.43)	2.39±1.15 (0.71–6.08)	2.38±1.05 (0.73–6.04)	2.31±1.22 (0.70–6.07)	**<0.0001**
ETV group		3.21±1.48 (1.27–7.62)	2.72±1.35 (0.96–7.04)	2.45±1.22 (0.81–6.26)	2.28±1.16 (0.73–6.04)	2.31±1.15 (0.71–6.06)	2.25±1.13 (0.70–6.09)	**<0.0001**

APRI: aspartate aminotransferase-to-platelet ratio index; TAF: tenofovir alafenamide; TDF: tenofovir disoproxil fumarate; ETV: entecavir; FIB-4: fibrosis index based on four factors; SD: standard deviation. Statistically significant values are denoted in bold.

Statistical analyses using the MIXED model followed by post-hoc analysis methods found no significant difference in the changes of APRI and FIB-4 scores over time according to the treatment (TAF, TDF, and ETV) (p=0.176).

### Virological and biochemical response

No difference was found in the statistical analysis regarding the time to achieve a virological response between the treatment groups (p=0.558). Similarly, the comparison analysis for the time to achieve a biochemical response showed statistical similarity between the TAF, TDF, and ETV treatment arms (p=0.705).

## DISCUSSION

All patients included in our study were HBeAg-negative. The patients in the TAF, TDF, and ETV arms were statistically similar in terms of age, gender, baseline HBV-DNA, ALT, AST, histological activity index (HAI), and fibrosis scores obtained by liver biopsy, APRI score, and FIB-4 indices. Additionally, the proportion of cirrhotic patients in each treatment arm was evenly distributed. We believe that these equal conditions contribute to the objectivity of our results in demonstrating the changes over time in non-invasive fibrosis indicators and the differences between the treatment arms. Improvements in the fibrotic burden during treatment with highly effective antiviral agents such as TAF, TDF, and ETV are known^13–15^. In the literature, the efficacy of tenofovir and ETV has been compared in many different studies. For example, in a meta-analysis, tenofovir and ETV were found to be the most effective oral antiviral agents for fibrosis improvement in the 1st year of CHB treatment for HBeAg-positive patients, with tenofovir being more effective in regressing fibrosis in HBeAg-negative patients^16^. In a similar study by Okan et al., the 4-year changes in APRI and FIB-4 scores with TDF, ETV, and lamivudine treatments were investigated, concluding that TDF and ETV were superior to lamivudine in improving liver fibrosis^14^. In a study conducted by Chon et al. on 3,277 patients, significant fibrosis regression was achieved with long-term antiviral treatment using ETV and TDF, as reflected by the APRI score and FIB-4 index^13^. In this study, despite higher baseline fibrosis burden in the ETV group, the fibrosis burden equalized at the end of the 1st year of antiviral treatment and remained stable between the 1st and 4th years. Some of our results are similar to the results of this study, and the difference in results may be due to the difference in the number of patients in the studies (3,277 versus 166). Additionally, in the study by Chon et al., the number of HBeAg-positive and HBeAg-negative patients is equal. Our study consists only of HBeAg-negative patients. In a study by Kim et al. involving 575 patients, the Ishak stage obtained by liver biopsy after 240 weeks of TDF treatment was compared with APRI and FIB-4 scores. It was reported that there was an inconsistency between the Ishak stage and APRI and FIB-4 levels in the majority of patients with advanced fibrosis or cirrhosis (81–89%), and in 71% of patients without fibrosis. The APRI score and FIB-4 index at the 240th week of treatment were found to underestimate the stage of fibrosis in patients undergoing liver biopsy. Therefore, the study concluded that the reduction in APRI or FIB-4 was not associated with fibrosis regression after 240 weeks of antiviral treatment^17^. İn our study, the decreases in APRI score and FIB-4 index were most pronounced in the first 2 years and then plateaued. Additionally, we compared the changes in fibrotic burden according to antiviral agents. It was concluded that the fibrotic burdens at the start of antiviral treatment were equal in patients treated with TAF, TDF, and ETV, and the patterns of fibrosis burden regression with these agents were statistically similar. A meta-analysis suggested that TDF is a better choice than ETV for chronic HBV patients because it has stronger viral suppression and a safety profile similar to ETV^18^. In a cohort study conducted in 2017, it was found that HBV DNA levels were similarly suppressed in both the ETV and TDF groups during a 12-month follow-up period, but the level of HBV DNA decrease was more pronounced in the TDF group compared to the ETV group in HBeAg-positive patients^19^. Another systematic review found a significant difference in undetectable HBV-DNA levels in the ETV group compared to the TDF group during the 3-month follow-up period, but there was no significant difference in the long-term period^20^. A recently published meta-analysis comparing TAF and TDF showed that there was no significant difference in viral suppression between the TAF and TDF groups after 12 months of treatment^21^. In our study, the mean times to virological clearance (HBV DNA levels falling below 50 IU/mL) in the TAF, TDF, and ETV groups were found to be 11.7±5.4 months, 12.83±0.7 months, and 12.43±5.9 months, respectively. In a meta-analysis published by Chen et al., it was shown that after 12 months of treatment, the rates of ALT normalization were higher in the TAF group compared to the TDF group, and the incidence of adverse reactions was lower in the TAF group.

## CONCLUSION

Significant fibrosis regression was observed in CHB patients using TAF, TDF, and ETV over the long term, as reflected by the APRI score and FIB-4 index. While the APRI score and FIB-4 index showed a continuous decline in the cumulative patient population and in the separate treatment arms during the first 2 years of treatment, these indicators remained stable between the 2nd and 5th years. The regression effects of TAF, TDF, and ETV, known as high-potency antivirals with a strong genetic barrier, on the fibrosis burden, and the changes in these effects over time, were found to be equivalent. Additionally, no superiority was found among these three drugs in terms of their effects on viral clearance and biochemical improvement in CHB patients. We believe that prospective studies with larger patient numbers, comparing the fibrosis regression reflected by the APRI score and FIB-4 index with the fibrosis burdens obtained by biopsy, are needed in CHB patients under treatment.

## Data Availability

The datasets generated and/or analyzed during the current study are available from the corresponding author upon reasonable request.
